# Synthesis and Determination of Chronic and Acute Thermal and Chemical Pain Activities of a New Derivative of Phencyclidine in Rats

**Published:** 2010

**Authors:** Abbas Ahmadi, Mohsen Khalili, Ramin Hajikhani, Leila Barghi, Farnaz Mihandoust

**Affiliations:** a *epartment of Chemistry, Faculty of Science, Islamic Azad University, Karaj Branch, Karaj, Iran.*; b *Department of Physiology, School of Medicine, Shahed University, Tehran, Iran.*

**Keywords:** Phencyclidine, Ketamine, 1-Tetralone derivatives, Tail immersion test, Formalin test, Acute and chronic pain

## Abstract

Phencyclidine (1-(1-phenylcyclohexyl) piperidine, PCP, I) and ketamine (2-*O*-chlorophenyl-2-methylaminocyclohexan, II) have shown analgesic effects. Some of its derivatives were synthesized and their biological properties have been studied. In this study, a new derivative of PCP, (1-[1-(3-methoxyphenyl) (tetralyl)] piperidine, PCP-OCH_3_-tetralyl, III) was synthesized and the acute thermal pain of this compound was determined using tail immersion test on rats and the results were compared with Ketamine and PCP.

The results indicated a marked anti-nociception 2-25 min after ketamine injection, but the analgesic effect remained for 40 min following PCP-OCH_3_-tetralyl application in tail immersion test. However, the data obtained from formalin test showed that the chronic anti-nociception effect of ketamine was higher than PCP and PCP-OCH_3_-tetralyl exhibited almost similar analgesic effect.

## Introduction

**Figure 1:**
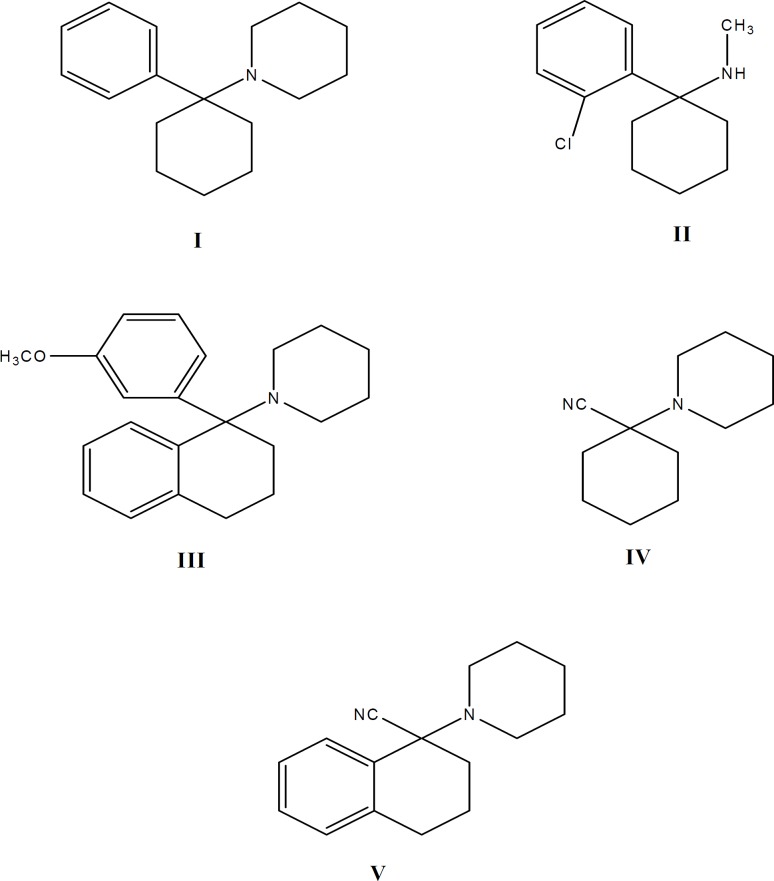
Structure formulas of PCP (**I**), Ketamine (**II**), PCP-OCH_3_-tetralyl (**III**) and Carbonitrile intermediates **IV **and **IV**

Phencyclidine (1-(1-phenylcyclohexyl) piperi- dine, CAS 956-90-1, PCP, I) is a semi-rigid molecule containing a cyclohexane ring with attached aromatic and piperidine rings (Figure 1). PCP and its analogues are highly potent and widely abused psychotomimetic drugs which influence the central nervous system. They display analgesic, stimulant, depressant and hallucinogenic effects due to specific binding sites in the brain (1). PCP binds to the *N*-methyl-D-asparate (NMDA) receptor complex and blocks NMDA-mediated gating of the calcium conductance channel (2). The analgesic effect of ketamine (2-*O*-chlorophenyl-2-methylaminocyclohexan, CAS 1867-66-9, II, Figure 1), another PCP analogue, was first described by Domino *et al*. in 1965 (3). Ketamine is a low-affinity, use-dependent, non-competitive antagonist of NMDA receptors (4-6). 

**Figure 2:**
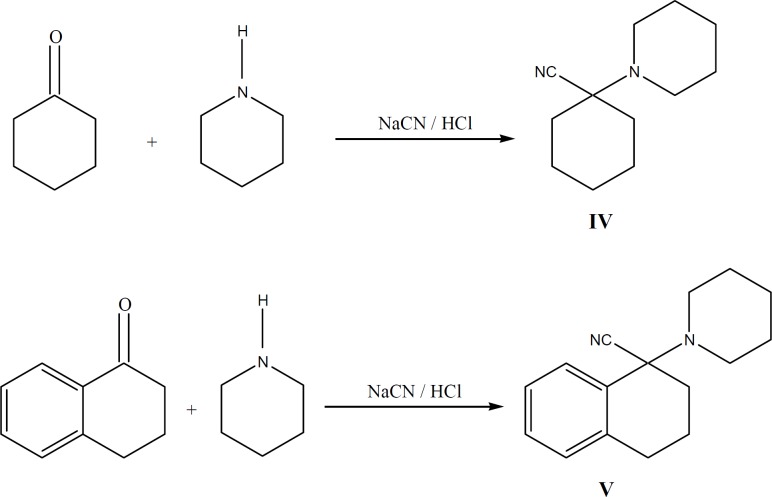
Synthesis of intermediates **IV **and **V**

**Figure 3:**
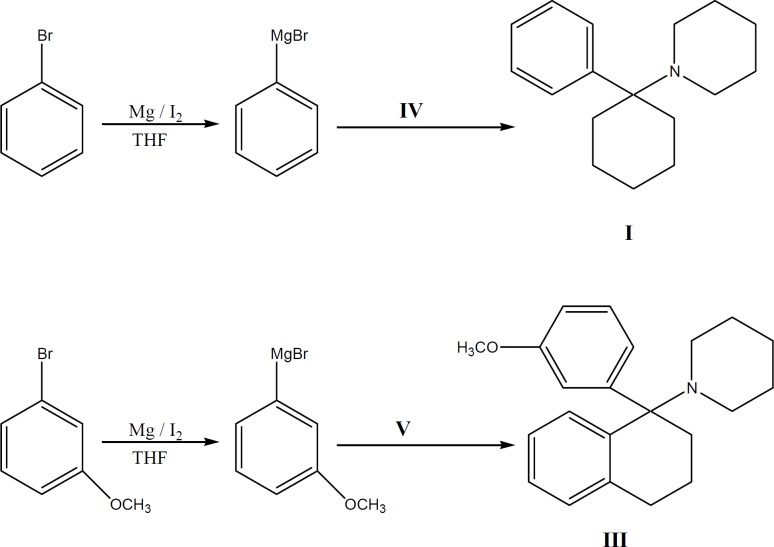
Synthesis of compoun ds**I**and**III**

**Figure 4:**
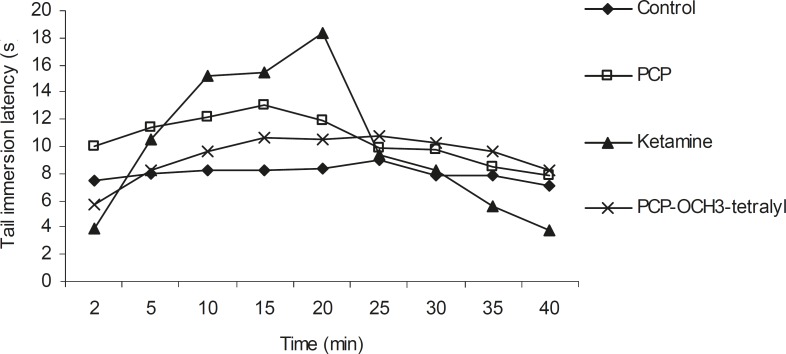
Mean tail immersion latency (s) in animals receiving ketamine, PCP and PCP-OCH_3_-tetralyl hydrochloride in saline (control). The tail immersion test was conducted 2, 5, 10, 15, 20, 25, 30, 35 and 40 min after drugs injection. Each point represents the mean ± SEM of tail immersion latencies (s) in 7-9 animals

**Figure 5:**
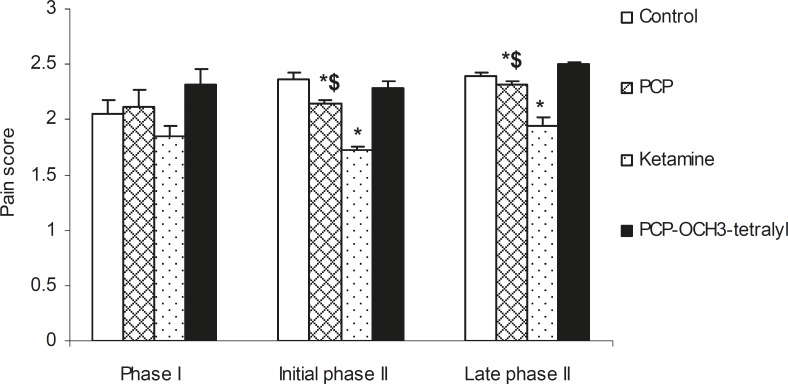
Comparison of the acute and chronic formalin pain in ketamine, PCP and PCP-OCH_3_-tetralyl hydrochloride animal groups compared with respect to control. Bars show the mean ± SEM of pain score. As indicated, administration of the drugs had no effect on acute pain (phase I), but the initial and late phases of chronic pain could be significantly reduced following the administration of PCP and especially ketamine. *n *= 12, * and $ (p < 0.05) show the difference with control and ketamine groups respectively

Recently, many analogues of phencyclidine have been synthesized (7-18) and their pharmacological activities have been studied. As part of our efforts to reach selective, non-competitive antagonists at the PCP binding site on NMDA receptor complex, we have prepared 1-[1-(3-methoxyphenyl) (tetralyl)] piperidine, (PCP-OCH_3_-tetralyl, III, Figure 1), as an analogue of PCP containing a methoxy group on the aromatic ring (*m*-position) and a phenyl group with cyclohexane ring (a conjugated cyclic ketone, 1-tetralone). We examined its analgesic effects on rats by tail immersion (as a model of acute thermal pain) and formalin (as a model of acute chemical and chronic pain) tests. The results have been compared with PCP and ketamine. It was anticipated that incorporation of methoxy group on the aromatic ring of the molecule will produce pronounced effect on electron distribution and dipole moments, due to high electron donating character of this group (7). Also it was anticipated that the incorporation of an extra aromatic and flat phenyl group on cyclohexane ring (a conjugated cyclic ketone, 1-tetralone) is able to reduce the conversion of the conformed isomers of the drug (19-21).

## Experimental

*Materials*

1-Tetralone [1, 2, 3, 4-tetrahydro-1-naphthalenone], Cyclohexanone, Piperidine, bromo benzene, magnesium turning, diethyl ether, 3-bromo anizole and all other chemicals, were supplied from Merck Chemical Co. (Darmstadt, Germany). Melting points (uncorrected) were determined using a digital electrothermal melting point apparatus (model 9100, Electrothermal Engineering Ltd., Essex, UK). ^1^H and ^13^C NMR spectra were recorded on a Bruker 300 MHz (model AMX, Karlsruhe, Germany) spectrometer (internal reference: TMS). IR spectra were recorded on a Thermo Nicolet FT-IR (model Nexus-870, Nicolet Instrument Corp, Madison, Wisconsin, U.S.A.) spectrometer. Mass spectra were recorded on an Agilent Technologies 5973, Mass Selective Detector (MSD) spectrometer (Wilmington, USA). Chromatographic column separations were performed over Acros silica gel (No.7631-86-9 particle size 35-70 micrometer, Geel, Belgium). Adult female Wistar rats (Pasteur Institute, Tehran, Iran), weighing 250 -300 g were used for pharmacological testing. 

*Methods*

*Synthesis of compounds (* Figure 1 *,* 2 *)*

*(1-(1-phenylcyclohexyl) piperidine (PCP) I*

This compound was prepared according to reported method (22) from 1-piperidinocy-clohexanecarbonitrile (IV) and phenyl magnesium bromide. The hydrochloride salt was prepared using 2-propanol and HCl and was recrystallized from 2-propanol (22). 

*1-Piperidinotetralylcarbonitrile V*

To a solution, containing 0.582 g (0.0068 mol) of piperidine in 0.253 g HCl (37%) and 1.36 g cold water, 1 g (0.0068 mol) 1, 2, 3, 4-tetrahydro-1-naphtalenone (1-tetralone) was added. Then 0.465 g KCN in 1.02 mL water, 50 mL ethanol and 0.1 g tetra-*n*-buthylammonium bromide (0.0003 mol) were added and stirred in ambient temperature (25^˚ ^C). The progress of reaction was controlled by TLC (7:3 ethyl acetate: *n*-Hexane). After one week no additional progress was observed and so the reaction was performed with chloroform (75 mL, 3 times). Then organic layer was separated, dried and concentrated. The oily residue obtained, was passed through a silica gel column using ethyl acetate: hexane (7:3) as the eluent to afford 1.13 g of V (69 % yield).

IR (KBr): 3066, 2941, 2560, 1454, 1436, 1324, 1287, 1225, 764 cm^-1^.

^1^H NMR (CDCl_3_) δ (ppm): 1.56 (6H, b, β and γ H of piperidine ring), 1.68 (2H, b, β H of cyclohexane ring), 1.85 (2H, b, α H of cyclohexane ring), 2.13 ( 4H, b, α H of piperidine ring), 2.74 ( 2H, b, γ H of cyclohexane ring), 6.82-6.95 (8H, m, ArH).

^13^C NMR (CDCl3) δ (ppm): 25.4, 26.2, 26.8, 31, 37.9, 46.7, 52.7, 117.7, 125.5, 128.1 and 139.2. 

MS: m/z (regulatory intensity): 240 [M]^+ ^(76), 241 [M+ H]^+^(15).

*1-[1-(3-methoxyphenyl) (tetralyl)] Piperidine III*

A solution containing 4 g (0.016 mol) of nitrile compound (V) in 10 mL of dry THF was added to a refluxing solution of (3-methoxylphenyl) magnesium bromide (Grignard reagent) (prepared from 24.77 g 3-bromoanisole and 3.075 g of Mg in 17 mL of dry ether), refluxed for 5 additional h in 65-67 ^o^C, left overnight at ambient temperature (25 ^o^C) and then poured into ice-NH_4_Cl. The organic layer was separated and washed with water and the base was neutralized with 10% H_2_SO_4_, washed with 20% NaOH, re-extracted with *n*-Hexane, dried and concentrated. The obtained oily residue was obtained, which was passed through a silica gel column using ethyl acetate: hexane (7:3) as the eluent to afford 2.28 g of III (42 % yield).

The hydrochloride salt of III was prepared using 2-propanol and HCl and was recrystallized from 2-propanol as an oily compound. 

IR (KBr): 3066, 2941, 1602, 1483, 1454, 1436, 1324, 1287, 1225, 764 cm^-1^.

^1^H NMR (CDCl_3_) δ (ppm): 1.51 (6H, b, β and γ H of piperidine ring), 1.62 (2H, b, β H of cyclohexane ring), 1.95 (4H, b, α H of cyclohexane ring), 2.24 (4H, b, α H of piperidine ring), 2.85 (2H, b, γ H of cyclohexane ring), 3.73 (3H, S, -OCH_3_), 6.93-7.01 (8H, m, ArH).

^13^C NMR (CDCl3) δ (ppm): 26.2, 27.5, 31.8, 44.8, 47.4, 56, 63, 111.6, 114, 120.2, 120.7, 125.8, 126.2, 128.8, 130, 139.3, 142.8, 144, 162.5. 

MS: m/z (regulatory intensity): 321 [M]^+^ (100), 322 [M+ H]^+^(7).

*Pharmacological methods*

Adult female Wistar rats (Pasteur Institute, Tehran), weighing 250-300 g at the begining of the experiment were randomly housed; three to four per cage, in a temperature-controlled colony room under light/dark cycle. Animals were given free access to water and standard laboratory rat chow (Pars Company, Tehran, Iran). All behavioral experiments were carried out between 11 a.m. and 4 p.m. under normal room light at 25 °C. All animals were injected by one of the investigator and evaluated by another. This study was carried out in accordance guidelines the policies provided in the guide for the care and use of Laboratory Animals (NIH) and those of the Research Council of Shahed University of Medical Sciences, Tehran, Iran.

*Tail immersion test*

Acute thermal pain is modeled by the tail immersion test (23-29). Two, 5, 10, 15, 20, 25, 30, 35 and 40 min after an intraperitoneal (IP) injection of drugs (6 mg/kg (8, 9, 15) which is under LD_50_ dosage limit of the drugs (9) ) (I, II, III) or an equivalent volume of saline (control), the rats (n = 7-9 in each group) were housed in an animal restrainer. Then, the terminal 5 cm of their tails were first submerged into room temperature water (22~24 °C) to check for an aversion to water and afterwards immersed in 52 °C water. The reaction time between immersing the tail and its removal out of the heated water was measured. A cut-off latency of 20 sec was employed to avoid damaging the tail.

*Formalin test*

Formalin test introduced by Dubuisson and Dennis (30) was used in our experiments. In this method, formaldehyde solution (50 μl, 2.5%) was injected subcutaneously into the plantar surface of hind paw. The animal was then placed in a plexiglass chamber (30×30×30 cm^3^) with a mirror at 45^o ^angle underneath, for accurate observation. In the treatment groups, the drugs (ketamine, PCP and PCP-CH_3_-tetralyl (III)) were administrated intraperitoneally 30 min prior to the formaldehyde injection. Prior to experiment, all animals were brought to the test chamber 5 times with 5 min interval in order to adapt them to the environment. The behavioral pain reactions due to formalin injection were detected and recorded for 1 h. The first 15 min, post-formalin injection is known as the early (I) or acute phase, and the period between 15-60 min is considered the second (II) or chronic phase. However, the chronic phase could be divided to initial (15-40 min) and late (40-60 min) periods. 

*Statistical analysis*

All data are expressed as means ± SEM. The mean latency of tail withdrawal (s) in tail immersion test and pain scores of formalin test in different groups was compared using one-way ANOVA followed by Tukey’s post-hoc test. We considered the probability of p < 0.05 as a significant difference.

## Results

*Chemistry*

Phencyclidine (I), and 1-[1-(3-methylphenyl) (tetralyl) piperidine (III) were synthesized by reaction of substituted Grignard reagents and carbonitrile compounds (IV, V). To obtain higher electron distribution and dipole moment properties (7), a methoxy group was substituted on the aromatic ring of the molecule (III) and to decrease the conversion of conformation isomers of the drug as of our previous results in tetraline series of PCP (9, 20, 21), we synthesized a new analogue of PCP with two additional groups on phenyl and cyclohexane rings of the molecule (III). Known procedures were applied for the synthesis of compounds I and IV with the appropriate modifications described previously (19, 22). 

Bromobenzene and its *m*-methoxy (II) derivative were reacted with magnesium to form Grignard reagents, which were then reacted with appropriate piperidinocyclohexanecarbonitrile (IV) and piperidinotetralylcarbonitrile (V). Reaction between the Grignard reagents and the carbonitriles were slow and incomplete. To overcome this barrier, molar ratio of Grignard reagents to carbonitriles was increased (19, 31). 

Spectroscopic data (IR, ^1^H and ^13^C NMR, Mass) confirmed the structure of compounds III and V. The melting points of known compounds could also confirm their identity. The purity of each compound was checked by TLC using ethyl acetate: *n*-hexane as the eluent.

*Pharmacology*

*General consideration*

Mortality, morbidity, irritability and other side effects could be associated with drugs administration. However, comparison of the motor coordination index (was measured by Rota-rod apparatus, Harvard, UK) indicated no significant differences between control and treatment rats.

*The analgesic activity of PCP (I), ketamine (II) and 1-[1-(3-methoxyphenyl) (tetralyl) piperidine (III) hydrochlorides with tail immersion and formalin test*

Intraperitoneal injection of PCP (I), Ketamine (II) and PCP-OCH_3_-tetralyl (III) hydrochloride (6 mg/kg) which were dissolved in saline, produced a significant analgesic effect in tail immersion test. Comparison of the analgesic effect of ketamine, PCP and PCP-OCH_3_-tetralyl revealed a pronounced anti-nociception in 2-25 min after ketamine injection, but this analgesic effect remained for 40 min following PCP-OCH_3_-tetralyl application (Figure 4). However, the data from formalin test showed that the chronic anti-nociception effect of ketamine was higher than that of PCP and PCP-OCH_3_-tetralyl which have nearly the same analgesic effect (Figure 5). The difference in the tail immersion latencies and pain scores were evaluated using analysis of variance method (ANOVA). 

Therefore it seems, strong electron donating properties of the methoxy group on *meta *position of phenyl ring and the incorporation of an extra aromatic and flat phenyl group with cyclohexane ring (a conjugated cyclic ketone, 1-tetralone) facilitated higher binding to NMDA receptor complex which could increase tail immersion latencies in comparison with ketamine and PCP with higher half life.

## Discussion

Electrophysiologic and binding studies revealed that when the channels are in the open or activated state, various antagonists of NMDA receptors, including phencyclidine, ketamine and MK-801 primarily binds to PCP-site (9, 32). Previous studies suggest that ketamine may interact with the NMDA receptor at two potentially distinct sites. The first one is one located within channel pore and the second one is associated with hydrophobic domain of the protein. The binding of the agonist to the receptor is assumed to modify the binding of ketamine to both sites (33). 

In the present study a new derivative of PCP (III) and its carbonitrile intermediate having changes in substitutions in its phenyl and cyclohexane rings was synthesized. Stronger analgesic effects of some of our synthesized derivatives of PCP with methyl, methoxy, hydroxyl groups on phenyl and cyclohexane rings was reported (8, 9, 15, 19) and higher electron distribution and dipole moments of methoxy group (7) as well as decreasing the conversion of conformation isomers of the drug were shown (9, 20, 21). Furthermore, due to our previous results in tetraline series of PCP (9), we synthesized a new analogue of PCP with two additional groups on phenyl and cyclohexane rings of the molecule (III) and we studied its analgesic effects. 

Comparison of the tail immersion and formalin tests data indicated that PCP-OCH_3_-tetralyl can diminish thermal but not chemical (formalin) acute pain. It demonstrated that perhaps different mechanisms are involved in thermal and chemical acute pain for such different responses. However, the long lasting effect for III compared to II, could be related to higher half-life of III compared to II. Perhaps, the impermeability of blood brain barrier (BBB) to PCP-OCH_3_-tetralyl could explain the non significancy in analgesic effect of III with regard to the marked analgesic effect of ketamine and PCP in initial and late phases of formalin-induced chronic pain (phase II). Chronic formalin pain (phase II) mediated by inflammatory mediators and CNS activation (34). 

## References

[1] Chen G, Ensor CR, Russell D, Bohner B (1959). The pharmacology of 1-(1-phenylcyclohexyl) piperidine HCl. J. Pharmacol. Exp. Ther.

[2] Honey CR, Miljkovic Z, McDonald JF (1985). Ketamine and phencyclidine cause a voltage-dependent block of responses to L-aspartic acid. Neurosci. Lett.

[3] Domino EF, Chodoff P, Corssen G (1965). Pharmacologic Effects of Ci-581 (A new dissociative anesthetic). Man. Clin. Pharmacol. Ther.

[4] Anis NA, Berry SC, Burton NR (1983). The dissociative anaesthetics, ketamine and phencyclidine, selectively reduce excitation of central mammalian neurones by N-methyl-aspartate. Br. J. Pharmacol.

[5] Tabrizian K, Najafi S, Belaran M, Hosseini-Sharifabad A, Azemi Hosseini A, Soodi M, Kazemi A, Kebriaeezadeh A, Sharifzadeh M (2010). Effects of selective iNOS inhibitor on spatial memory in recovered and non-recovered ketamine induced-anesthesia in wistar rats. Iranian J. Pharm. Res.

[6] Monaghan DT, Bridges RJ, Cotman CW (1989). The excitatory amino acid receptors: their classes, pharmacology, and distinct properties in the function of the central nervous system. Annu. Rev. Pharmacol. Toxicol.

[7] Ahmadi A, Mahmoudi A (2006). Synthesis with improved yield and study on analgesic effect of 2-methoxyphencyclidine. Arzneim-Forsch-Drug Res.

[8] Al-deeb OAA (1994). Synthesis and analgesic activity of new phencyclidine derivatives. Arzneim-Forsch-Drug Res.

[9] Ahmadi A, Mahmoudi A (2005). Synthesis and biological properties of 2- hydroxy-1-(1-phenyltetralin) piperidine and some of its intermediates as derivatives of phencyclidine. Arzneim-Forsch-Drug Res.

[10] Shimoyama N, Shimoyama M, Inturrisi CE (1996). Ketamine attenuates reverses morphine tolerance in rodents. Anesthesiol.

[11] Fuman B, Aldinger G, Fauman M, Rosen P (1976). Psychiatric Squeal of phencyclidine abuse. Clin. Toxicol.

[12] Al-deeb OAA (1996). New analgesic derived from the phencyclidine analogue thiencyclidine. Arzneim-Forsch-Drug Res.

[13] Ogunbadeniyi AM, Adejare A (2002). Syntheses of fluorinated phencyclidine analogs. J. Fluo. Chem.

[14] Ahmadi A, Shafiezadeh M, Fathollahi Y (2005). Synthesis with improved yield and study on analgesic effect of 2-hydroxyphencyclidine. Arzneim-Forsch-Drug Res.

[15] Kamenka JM, Ung MSN, Herrmann P (1979). Determination conformationnelle de derives de la phencyclidine en vue d’une correlation acture-active. Eur. J. Med. Chem. Therapeutica.

[16] Kamenka JM, Chiche B, Goudal R, Geneste P, Vignon J, Vincent JP (1982). Chemical synthesis and molecular pharmacology of hydroxylated 1-(1-phenylcyclohexyl) piperidine derivatives. J. Med. Chem.

[17] Itzhak Y, Kalir A, Weissman BA, Cohen S (1981). New analgesic drugs derived from phencyclidine. J. Med. Chem.

[18] Ahmadi A, Khalili M, Abbassi S, Javadi M, Mahmoudi A, Hajikhani R (2009). Synthesis and study on analgesic effects of 1-[1-(4-methylphenyl) (cyclohexyl)] 4-piperidinol and 1-[1-(4-methoxyphenyl) (cyclohexyl)] 4-piperidinol as two new phencyclidine derivatives. Arzneim-Forsch-Drug Res.

[19] Darvich MR, Zonoozi A (1993). Preparation of the phencyclidine analogues (part II). Iran. J. Chem. & Chem. Eng.

[20] Kaorv H (1974). O-H stretching absorption and conformation of the β-methyl derivatives of 1-tetralol, 4-chromanol, 4-thiochromanol, and 4-thiochromanol 1, 1-dioxide. Bull. Chem. Soc. Jpn.

[21] Nobuo M, Mitsuo Y, Yoshihiro A, Yojiro T (1971). Intramolecular hydrogen bonds. XVI. Preferable conformation of 1-tetralols. Bull. Chem. Soc. Jpn.

[22] Geneste P, Kamenka JM, Dessapt P (1980). Method for stereoselective production of substituted cyclohexylcyanhydrines. Bull. Soc. Chim. Fr.

[23] Ramabadran K, Bansinath M, Turndorf H, Puig MM (1989). Tail immersion test for the evaluation of a nociceptive reaction in mice. Methodological considerations. J. Pharmacol. Methods.

[24] Hamura H, Yoshida M, Shimizu K, Matsukura T, Suzuki H, Narita M (2000). Antinociceptive effect of the combination of pentazocine with morphine in the tail-immersion and scald-pain tests in rats. Jpn. J. Pharmacol.

[25] Molina N, Bedran-de-Castro MT, Bedran-de-Castro JC (1994). Sex-related differences in the analgesic response to the rat tail immersion test. Braz. J. Med. Biol. Res.

[26] Ibba M, Schiavi S, Abbiati G, Testa R (1987). Effects of oral administration of nortriptyline, imipramine and citalopram on morphine hot-plate and tail-immersion analgesia in rats. Bull. Chim. Farm.

[27] Sewell RD, Spencer PS (1976). Antinociceptive activitiy of narcotic agonist and partial agonist analgesics and other agents in the tail-immersion test in mice and rats. Neuropharmacol.

[28] Padi SSV, Kulkarni SK (2008). Minocycline prevents the development of neuropathic pain, but not acute pain: Possible anti-inflammatory and antioxidant mechanisms. Eur. J. Pharm.

[29] Ibrahim MM, Rude ML, Stagg NJ, Mata HP, Lai J, Vanderah TW, Porreca F, Buckley NE, Makriyannis A, Malan JTP (2006). CB2 cannabinoid receptor mediation of antinociception. Pain.

[30] Shams J, Sahraei H, Faghih-Monzavi Z, Salimi SH, Fatemi SM, Pourmotabbed A, Ghoshooni H, Kamalinejad M (2008). Effects of Papaver rhoeas extract on the tolerance development to analgesic effects of morphine in mice. Iranian J. Pharm. Res.

[31] Jones LA, Beaver RW, Schmoeger TL (1981). Ort JF, Leander JD. Isolation, Identification and synthesis of compounds cosynthesized in the preparation of phencyclidine. J. Org. Chem.

[32] Parsons CG, Gibbens H, Magnago TSL (1988). At which ‘sigma’ site are the spinal actions of ketamine mediated?. Neurosci. Lett.

[33] Qian J, Brown SD, Carlton SM (1996). Systemic ketamine attenuates nociceptive behaviors in a rat model of peripheral neuropathy. Brain Res.

[34] Bonnefont J, Alloui A, Chapuy E, Clottes E, Eschalier A (2003). Orally administered paracetamol does not act locally in the rat formalin test: evidence for a supraspinal, serotonin-dependent antinociceptive mechanism. Anesthesiol.

